# Simulation-Based Optimization of a Multiple Gas Feed Sweetening Process

**DOI:** 10.1021/acsomega.1c05193

**Published:** 2022-01-13

**Authors:** Weixuan Zhu, Haotian Ye, Yang Yang, Xiong Zou, Hongguang Dong

**Affiliations:** School of Chemical Engineering, Dalian University of Technology, Dalian 116024, China

## Abstract

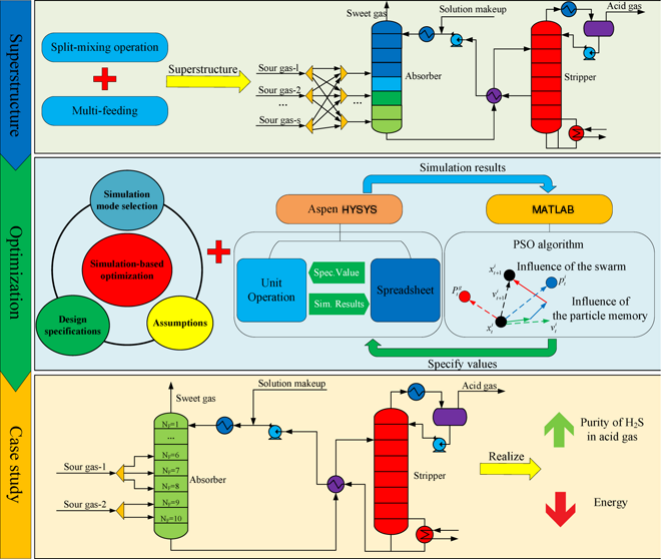

For an energy-intensive sweetening process, it is common that sour gases from different sources are sent to a single sweetening plant in industries. In our previous work, a multiple gas feed sweetening process was proposed, which can simultaneously improve the purity of H_2_S and reduce the energy consumption of the plant. This study aims to develop the superstructure of that process and use a simulation-based optimization framework with Aspen HYSYS as the process simulator and particle swarm optimization algorithm as the optimizer. In addition, by taking full advantage of the robustness of the built-in algorithm of the simulator, the convergence of the model is improved; meanwhile, simplification of the process and reduction of the optimization time are accessible with the proposed design specifications and assumptions. For a convergence-difficult column, a stepwise convergence adjustment was used to ensure their convergence. Based on this, the robustness and effectiveness of the method is proven through a case study, and it can also provide guidance for model selection, process simplification, and optimization of the same type of absorption process.

## 1 Introduction

In industry, sweetening is an indispensable process to protect the environment, prevent pipeline corrosion and catalyst deactivation, etc. Several methods have been applied in sweetening plants, such as absorption through a column or membrane contactor,^1−3^ adsorption,^4−6^ cryogenic separation,^7^ and membrane separation,^8^ among which the chemical absorption by amine solution is more widely used. Although the sweetening process is well-matured, the energy consumption of the plant is very high. As far as we know, steam consumption of the plant accounts for more than 10% of that of the entire refinery, so the research and improvement of the process have been going on.

The improvement of the process is mainly concentrated in three aspects, namely, the optimization of the process configuration, the industrialization of other technologies mentioned above or the development of hybrid technologies,^9−11^ and the development of new solutions.^12−14^ Among them, the improvement of the process configuration can be directly applied to the optimization of the plant. Ahn et al.^15^ evaluated 10 different configurations of sweetening processes and the most representative ones are the absorber intercooling configuration and split-solvent configuration. The absorber intercooling configuration can remove the absorption heat through the intercooler and effectively promote the absorption of acid components. The split-solvent configuration obtains a semilean solvent through partial separation or flash, which significantly reduces the amount of the lean solvent. All of the abovementioned configurations can ultimately reduce the energy consumption of the plant. Antonini et al.^16^ proposed a modified version based on the split-solvent configuration. The semilean solvent is split twice to obtain a cold semilean solvent and enters the top of the desorber, which can partially replace the reflux of the desorber, resulting in lower consumption of the plant. In addition, there are various schemes^17,18^ to increase the H_2_S/CO_2_ ratio in the feed gas by recycling part of the acid gas back to the raw materials so that the content of H_2_S in the acid gas will be increased, thereby reducing the energy consumption of the downstream sulfur recovery plant.

**Figure 1:**
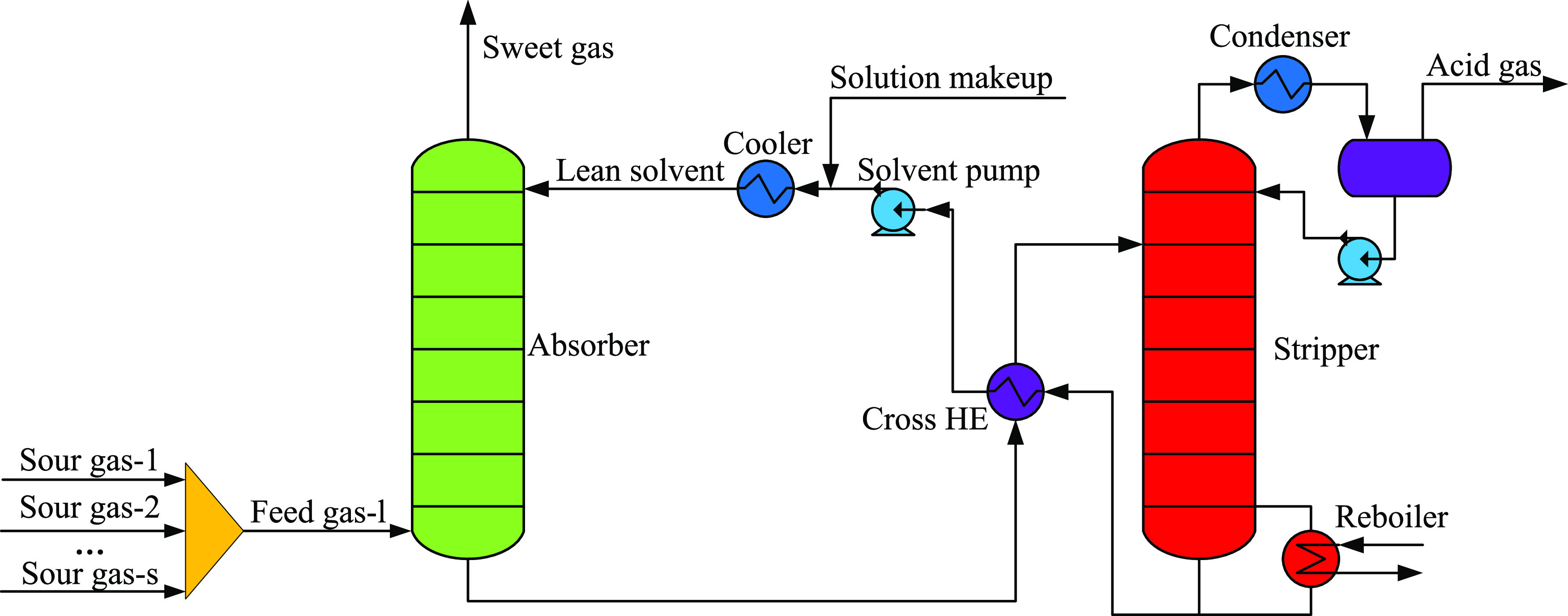
Scheme of the conventional sweetening process with multiple sour gases.

However, there is only one feed gas in the current process configuration, but whether it is petrochemical, coal chemical, or natural gas chemical, the source of sour gases is always diverse. For example, there are different processing plants, such as catalytic cracking, hydrocracking, etc., producing sour gases, which makes the sweetening plant always treat multiple streams of sour gases from different sources. Since there is no study specifically aimed at the sweetening process of multiple sour gases, the conventional process^19^ (Figure 1) is usually used, that all of the sour gases are premixed into one total feed stream and sent to the bottom of the absorber. But with the generation of reaction heat and the absorption of other acid components such as carbon dioxide, the mass transfer of H_2_S in the lower section of the absorber is poor, while the conventional process lacks adjustment means to improve the mass transfer. If the flow rate and composition characteristics of each sour gas are made full use of, the temperature and concentration distribution in the absorber can be optimized through preallocation and multifeeding operation, and ultimately, the mass transfer of H_2_S can be strengthened so as to improve the purity of H_2_S in acid gas and reduce energy consumption. Therefore, in the previous work, a novel multiple gas feed sweetening process was proposed.

Since ionic reactions are involved in the sweetening process, a complex thermodynamic model is used to accurately calculate the process of ion dissolution and ionization. Moreover, the use of an equilibrium-based model for the absorber and stripper in the sweetening process will bring great deviations,^20^ and a more accurate rate-based model is required. Therefore, its optimization is a nonlinear extremum problem with many variables and constrains. Sensitivity analysis or sequential iteration methods cannot very easily handle the multivariable optimization problem; as the iteration step size decreases or optimization variables increase, the optimization scale increases exponentially. The disadvantage of the conventional mathematical programming methods is the difficulty in convergence of the rigorous model of the distillation column.^21^ The shortcoming is the use of vapor–liquid equilibrium conditions on all trays of the column, which produces numerical problems as a result of the convergence of the equilibrium equation. However, the use of a more complex rate-based model will undoubtedly make convergence more difficult. In addition, there are many difficulties in mathematical programming, which require different experts in the fields of programming, modeling, and optimization to solve different types of problems, such as model initialization, debugging, and improving the accuracy of the results.^22,23^

Effective solutions to the above problems are surrogate-based optimization methods^24,25^ and simulation-based optimization methods. However, as the prediction accuracy and the number of variables increase, the training of the surrogate model will become complicated and time-consuming. Especially for the multiple gas feed sweetening process, small changes will affect the mass transfer and reaction in the absorber, so a high-fidelity model is essential for the optimization. There have been a series of studies using simulation-based optimization methods, such as the works of Oh et al.,^26^ Shirmohammadi et al.,^27^ and Ledezma-Martínez et al.,^28^ which optimize the CO_2_ capture process, the CO_2_ recovery unit utilizing the absorption refrigeration system, and the crude oil distillation system with a preflash unit, respectively. These processes are well known to have models that are relatively complex, and they lack shortcut models with high accuracy. The use of simulation-based optimization methods to optimize these processes has proven to be a very effective tool for process improvement and optimization. Table 1 lists some literature studies of this method in the past 5 years. In this work, considering that the sweetening process involves two components, i.e., obtaining a high-fidelity model for chemical absorption and also effectively optimizing it, a simulation-based optimization method is also used. The process simulator integrated with an external optimizer is used in simulation-based optimization. Commercial process software, e.g., Aspen HYSYS, uses a modular method to construct the flowsheet, and its built-in algorithm can perform the initialization and convergence of the module very well. The external optimizer can use metaheuristic algorithms instead of gradient-based algorithms,^37^ which can avoid the trouble of being unable to obtain accurate derivative information due to the influence of numerical noise.

**Table 1:** Literature Studies of the Simulation-Based Optimization Method in the Past 5 Years

softwares	algorithm	research content	refs
Unisim + MATLAB	GA	optimization of a superstructure including CO_2_ capture configuration and four different types of structural modifications	(26)
Aspen HYSYS + MATLAB	GA	optimization of post-combustion CO_2_ recovery unit utilizing the absorption refrigeration system	(27)
Aspen HYSYS + MATLAB	GA	optimization of crude oil distillation systems with preflash units	(28)
Aspen HYSYS + MATLAB	PSO	optimization and comparison of cryogenic distillation and membrane separation in helium extraction processes integrated with nitrogen removal units	(29)
Aspen HYSYS + MATLAB	PSO	optimization and comparison of four distillation-based configurations in propylene–propane separation	(30)
Chemkin Pro + Aspen HYSYS + MATLAB	GA	multiobjective optimization of sulfur recovery units using a detailed reaction mechanism	(31)
Aspen HYSYS + MATLAB	GA/PSO	optimization of a whole green-field saturated gas plant	(32)
Aspen Plus + MATLAB	SADDE	optimization and comparison of double-effect distillation and self-heat recuperation technology in ethylbenzene/styrene separation	(33)
Aspen HYSYS + MATLAB	GA	multiobjective optimization of the natural gas liquefaction process	(34)
Aspen Plus + MATLAB	NSGA-II	multiobjective optimization for the operation of the product separation process in a methanol to propylene plant	(35)
Aspen HYSYS + MATLAB	PSO	optimization of the heavy hydrocarbon removal process that reduces the heating value of LNG to meet desired specifications	(36)

The purpose of this work is to apply a simulation-based optimization strategy for optimizing operating conditions for the multiple gas feed sweetening process through coupling Aspen HYSYS and MATLAB with PSO algorithms. The novelty of this work is to make full use of the robustness of the Aspen HYSYS built-in algorithm, simplify the process through design specifications and reasonable assumptions, and greatly reduce the simulation time, thereby reducing the optimization time. In addition, for high-fidelity models that are prone to convergence problems, we use a strategy of using MATLAB to control the simulation sequence and a stepwise adjustment method to adjust the convergence-difficult column for better convergence. This work not only developed an optimization framework for the multiple gas feed sweetening process but also provided guidance for model selection, process simplification, and optimization of the same type of absorption process.

## 2 Process Description

**Figure 2:**
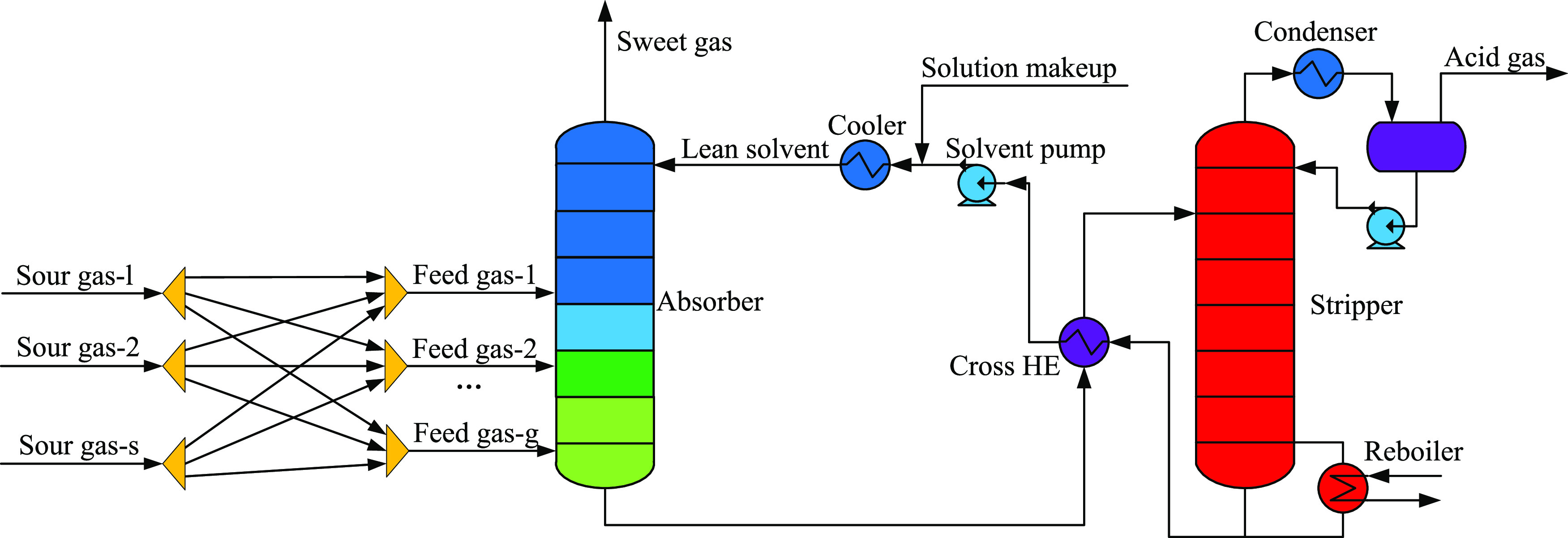
Scheme of the multiple gas feed sweetening process.

A schematic diagram of the multiple gas feed sweetening process is provided in Figure 2. A total of *s* sour gases are assumed to be sent to the plant. The preallocation of sour gases by means of split-mixing operation along with multiple feeding was proposed to obtain a total of *g* feed gases with their optimal flow rates, compositions, and feed stages. Each feed gas is sent to the absorber for sweetening to obtain sweet gas and the rest of the process is the same as the conventional process.

**Figure 3:**
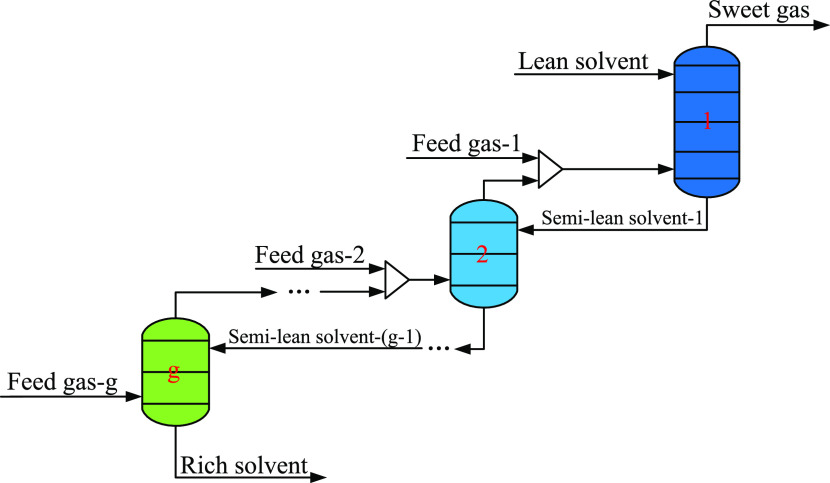
Equivalent absorber network model.

The equivalent absorber network model is shown in Figure 3. According to the feed stage of each feed gas, the absorber is divided into a total of *g* column sections. The feed gas of column section 1 is absorbed with the lean solvent, which is the same as the conventional single gas feed absorber, and the feed gas of the remaining column sections is absorbed with the semilean solvent of different concentrations. Based on the above operations, the absorption rates of H_2_S and CO_2_ in each column section can be effectively controlled. From the previous work,^38^ the feed gas with a higher CO_2_/H_2_S ratio should be sent to the upper column section, which can effectively reduce the absorption amount of CO_2_ and improve the purity of H_2_S in the acid gas. Moreover, through the optimization of the temperature and concentration distribution in the absorber, the mass transfer of H_2_S from column section 2 to *g* can be strengthened so that the concentration of H_2_S in the gas phase, which is sent to the column section 1, is lower than that of the conventional process. Under the same sweetening effect, both the amount of lean solvent and the energy consumption of the stripper can be reduced. Therefore, through the preallocation of sour gases, the temperature, composition, and feed stage of each feed gas are optimized so that the multiple gas feed sweetening process can improve the purity of H_2_S in acid gas, reduce energy consumption, and achieve simultaneous optimization of mass and energy.

## 3 Process Modeling Framework

### 3.1 Modeling of the Absorption–Desorption Process

Since in the MDEA–H_2_O–H_2_S–CO_2_ system, the absorption and desorption are significantly affected by the mass transfer and heat transfer, the use of the equilibrium-based model will lead to large errors.^13^ It is necessary to use a high-fidelity model based on the rate-based model and consider the ionic characteristics to simulate the actual process.

#### 3.1.1 Chemical Reaction

Since MDEA, as a tertiary alkanolamine, cannot directly react with CO_2_, other reactions are on the basis of proton transfer, which can be regarded as instantaneous compared to the mass transfer rate.^39^ Therefore, the reactions in the liquid phase can be divided into kinetically controlled reactions and equilibrium-controlled reactions^40^

(1) kinetically controlled reactions
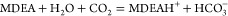
R1
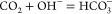
R2
(2) equilibrium-controlled reactions

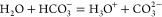
R3
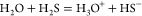
R4
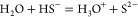
R5
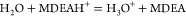
R6
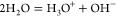
R7

For the calculation of chemical reactions, the equilibrium-controlled reactions R3–R7 can be calculated with the reaction equilibrium constant eq 1, which is calculated through Gibbs free energy eq 2
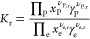
1
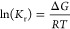
2

For the kinetically controlled reactions R1 and R2, the calculation of rate constants of forward and reverse reactions is of great importance. The reaction rates of R1 and R2 are presented in eqs 3 and 4. The kinetic parameters for the forward reactions of R1 and R2 are taken from the work of Rinker et al.^41^ and Pinsent et al.^42^ The rate constants of each kinetically controlled reaction are presented in eqs 5–8
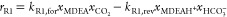
3
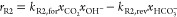
4
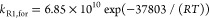
5
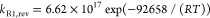
6
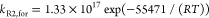
7
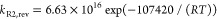
8

#### 3.1.2 Rate-Based Mass Transfer Model

**Figure 4:**
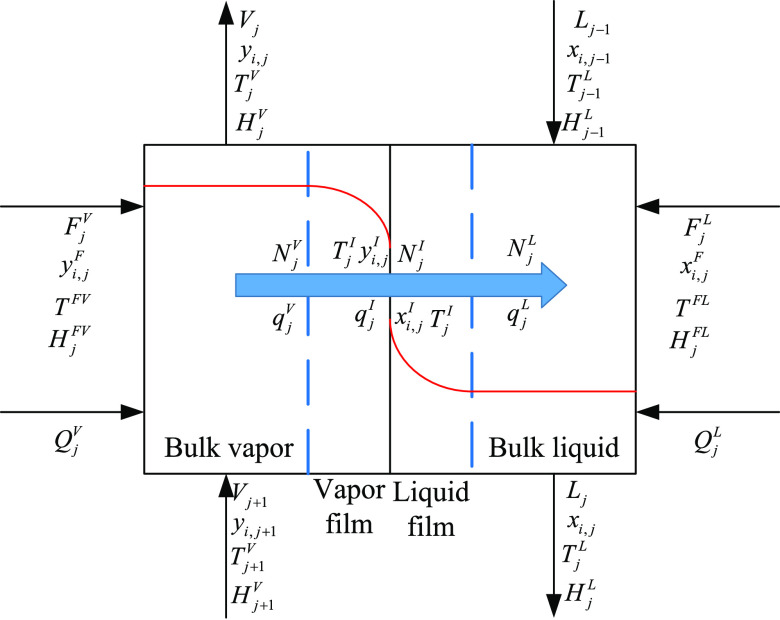
Schematic diagram of the two-film theory.

In this work, the two-film theory method^43^ was employed to establish the rate-based model. The schematic diagram of the two-film theory is shown in Figure 4, and the main assumptions^44,45^ for the model development are summarized below:

(1) It is assumed that there is a phase interface where the phase equilibrium is reached.
(2) The resistances of mass transfer and heat transfer are located in the boundary layer on the gas and liquid side, respectively.
(3) The two films are stagnating, so only diffusive mass transfer is considered.
(4) The bulk phases are ideally mixed with uniform concentrations and temperatures.
(5) The interfacial area of mass transfer and heat transfer is the same.
(6) Flow is one-dimensional and the variation of concentration and temperature in the radial direction is negligible.
(7) The column is under adiabatic mode of operation.

The equations of the entire model can be divided into three parts: governing equations, transfer equations, and auxiliary equations.

##### 3.1.2.1. Governing Equations

Similar to the equilibrium-based model, the governing equations of the rate-based model also contain material balance, energy balance, phase equilibrium, and summations, and the reaction process is considered in the material and energy balance. However, the difference is that the amount of mass transfer and heat transfer must be taken into consideration for the rate-based model.

The calculations of material balance for bulk liquid, bulk vapor, liquid film, and vapor film are displayed in eqs 9–12 as follows
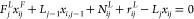
9
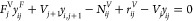
10
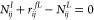
11
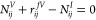
12

The calculations of energy balance for bulk liquid, bulk vapor, liquid film, and vapor film are displayed in eqs 13–16 as follows
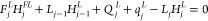
13
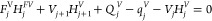
14
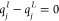
15
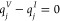
16

The calculation of phase equilibrium at the interface is displayed in eq 17 as follows
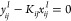
17

The summations of the components in bulk liquid, bulk vapor, liquid film, and vapor film are displayed in eq 18 as follows
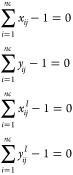
18

##### 3.1.2.2. Transfer Equations

For the absorption process involving chemical reactions, the conventional methods introduce the concept of the enhancement factor based on several simplifications^46,47^ to calculate the chemical absorption process. However, in the sweetening process, there are complex parallel and consecutive reactions in the liquid phase, which makes it difficult to accurately describe the process only by the enhancement factors. Therefore, the characteristics of the ions are considered in the model to directly describe the absorption process instead of enhancement factors.^48^

(1) Mass transfer equation of liquid phase:
  contrary to the equilibrium-based model, the calculation of interfacial flux in the rigorous rate-based model is required. The Maxwell–Stefan theory, a rigorous multicomponent mass transfer theory^49^ with binary mass transfer coefficients, was used to calculate multicomponent mass transfer coefficients and mass transfer rates of each component between the vapor and liquid phases.
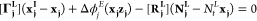
19where
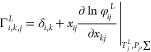
20
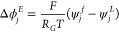
21
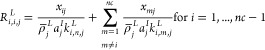
22
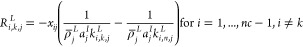
23
  The introduction of the electrical potential requires an additional equation to assure that the liquid phase is electroneutral everywhere
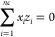
24
(2) Mass transfer equation of gas phase:
  the mass transfer equation of the gas phase is similar to that of the liquid phase, the only difference is that there are no ions in the gas phase and ionic characteristics need not be considered.
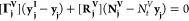
25where
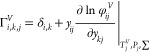
26
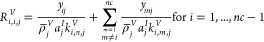
27
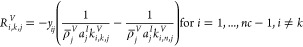
28
(3) Heat transfer equation of liquid phase:
  the heat flux through the liquid film comprises the conductive and convective terms:
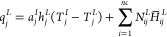
29
(4) Heat transfer equation of gas phase:

The heat transfer equation of the gas phase is the same as that of the liquid phase.
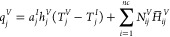
30

##### 3.1.2.3. Auxiliary Equations

In this work, the well-known and accepted correlations, which have been fitted to experimental measurements from absorption and distillation columns of laboratory and pilot plant, are used. According to the applicable conditions of each correlation, the AICHE,^50^ the Scheffe and Weiland,^51^ the Chilton and Colburn,^43^ and the Bennett, Agrawal, and Cook^52^ correlations are used to calculate binary mass transfer coefficients, interfacial area, heat transfer coefficients, and liquid holdup, respectively.

### 3.2 Selection of Simulation Mode

All of the above equations are built-in equations of rate-based distillation in Aspen HYSYS, and the sweetening process model is also simulated through Aspen HYSYS v.11 with the Acid Gas property package. The equations of the rate-based model are more than that of the equilibrium model and containing a lot of differential and high power equations, which makes calculations more difficult. Aspen HYSYS uses an efficient, Newton-based simultaneous correction approach in which the full set of equations is solved using Newton’s method, and the equilibrium-based model is used to obtain the initial guess. In addition, there are two rate-based modes for the simulation of the absorber and stripper: efficiency and advanced mode. The advanced mode uses the above equations and through the Maxwell–Stefan theory to rigorously calculate the heat and mass transfer rates without thermal or chemical equilibrium assumption between the vapor and liquid phases for each stage. The efficiency mode uses the rigorous rate-based calculations with some simplifications to calculate the Murphree efficiencies for H_2_S and CO_2_, which are further applied to solve the column based on the conventional equilibrium-based method. The simplifications in efficiency mode are that it does not account for resistance to heat transfer and assumes the liquid phase is in chemical equilibrium.

Due to the relatively high operating temperature of the stripper, the heat transfer and reactions are fast, which conforms to the assumptions of the efficiency mode. Therefore, the efficiency mode is suitable for simulating the stripper and its built-in algorithm can ensure good convergence even if various types of design specifications are defined. Since the mass and heat transfer have significant impacts on the absorber, the use of advanced mode can ensure the accuracy of the simulation results. Detailed comparison of the two simulation modes and the accuracy of the simulation results are provided in the Supporting Information (see Section S1).

## 4 Optimization-Simulation Methodology

### 4.1 Objective Function

Economic benefit was taken as the objective function for the conventional process and proposed process optimization (eqs 31–34)
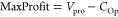
31
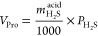
32
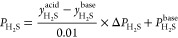
33
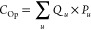
34

Due to the negligible changes in equipment cost for different configurations, the economic benefit is considered to be the only difference between the product value (*V*_pro_) and operating cost (*C*_op_).

The product value is calculated based on the price (*P*_H_2_S_) and flow rate (*m*_H_2_S_^acid^) of H_2_S in acid gas. The price of H_2_S is to reflect the impact of H_2_S with different purity on the fixed investment and operating costs of the downstream sulfur recovery unit. Thus, it was estimated based on that of the basic purity (*y*_H_2_S_^base^) with known price (*P*_H_2_S_^base^) and the additional value (Δ*P*_H_2_S_). The *y*_H_2_S_^base^, *P*_H_2_S_^base^, and Δ*P*_H_2_S_ are all derived from the literature,^38^ which are 70%, 142.86 $/t, and 0.72 $/t, respectively.

The operating cost is calculated by the duties (*Q*_u_) of the condenser and reboiler and the price (*P*_u_) of the utilities used. For cooler and pumps, their operating cost change is trivial and negligible compared to that of the condenser and reboiler. The information of utilities introduced is shown in Table 2. Low-pressure steam is selected as the hot utility for the reboiler, while cooling water is employed as a cold utility for the condenser.

**Table 2:** Information of Utilities

items	pressure (kPa)	inlet temperature (°C)	exit temperature (°C)	price ($/kJ)
LP steam	1137.6	184.2	184.2	1.08 × 10^–5^
cooling water	345	23.8	35	6.87 × 10^–7^

For the multiple gas feed sweetening process, various feed gases with appropriate flow rate, composition, and feed stage can be obtained by optimizing the split ratio of each sour gas to different feed stages. In addition, it is necessary to adjust the circulating flow rate of the lean solvent to ensure the quality of sweet gas. Therefore, there are two types of decision variables that need to be optimized.

(1) Split ratio of each sour gas to different feed stages (SR)
(2) Circulating flow rate of the lean solvent (*F*_s_)

The process design parameters (output from HYSYS) included in the objective function are:

(1) Flow rate of H_2_S in acid gas (*m*_H_2_S_^acid^)
(2) Purity of H_2_S in acid gas (*y*_H_2_S_^acid^)
(3) Duties of the condenser and reboiler (*Q*_con_ and *Q*_reb_)
(4) Content of H_2_S in sweet gas (*y*_H_2_S_^sweet^)

### 4.2 Constraints

Constraints include explicit constraints and implicit constraints. Explicit constraints are to ensure that the content of certain components in the sweet gas is within the specified range, which appears in the objective function in the form of a penalty function (see eq 35) to constrain solutions.
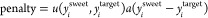
35where
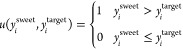
36

The implicit constraints, which were applied based on surrounding environmental conditions, process design guarantee, specifications, and common standard practice in the field, were imposed on the Aspen HYSYS model through a large list of “design specifications”.^30^ These implicit constraints are as below.

(1) Specify the condensation temperature at the top of the stripper: since the temperature of cooling water is 30 °C, the condensation temperature is 40 °C to keep the minimum temperature approach to 10 °C, which is often used in industry and literature studies.^30^ In this way, the content of water in the acid gas can be reduced as much as possible to meet the transportation and process requirements of the downstream plant.^53^
(2) Specify the separation effect of the stripper: in this work, the content of H_2_S in the lean solvent at the bottom of the stripper is used as an indicator of the separation effect. Through this design specification, the content of H_2_S in the lean solvent can be maintained unchanged during circulation so that the process is better converged.

Through the above design specifications, the freedom degree of the stripper is zero. Aspen HYSYS can automatically adjust the reflux ratio and overhead vapor rate of the stripper to meet the constraints, and its built-in algorithm can effectively ensure the column convergence, which can avoid the reflux ratio and overhead vapor rate as decision variables, reducing the complexity of optimization.

### 4.3 Computational Time Reduction

**Figure 5:**
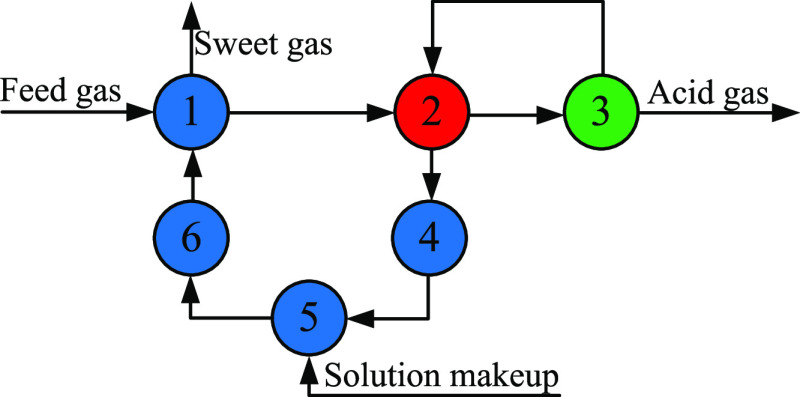
Information flow diagram of the sweetening process (1—absorber, 2—cross heat exchanger, 3—stripper, 4—solvent pump, 5—solution makeup, and 6—cooler).

In each iteration of the optimization, almost all of the computational time (>99.0%) is spent on running the simulation,^54^ especially when there are loops in the process, where convergence is time-consuming. The information flow diagram of the process is shown in Figure 5, and there are two loops in the whole system according to node 2.

To simplify the simulation difficulty and reflect the realistics of the process, the following assumptions are considered in the simulation:

(1) At the design stage, it is appropriate to replace the cross heat exchanger with a heater and a cooler, so the rich solvent enters a heater and finally to the top of the stripper. Because the temperature of the lean solvent at the bottom of the stripper is generally between 110 and 120 °C, to ensure the minimum heat transfer temperature difference, the temperature of the rich solvent sent to the stripper is specified as 100 °C.
(2) When the content of H_2_S in the lean solvent at the bottom of the stripper is fixed, the content of CO_2_ changes little during the optimization process so that its influence on the process is negligible. Therefore, the content of CO_2_ in the lean solvent is specified equal to that of the conventional process.

**Figure 6:**
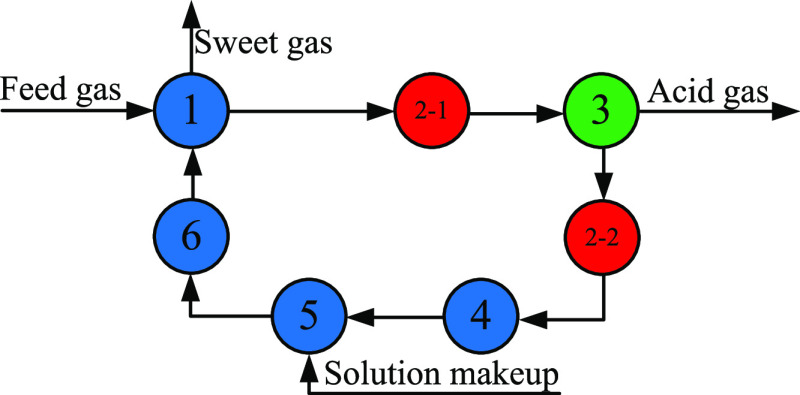
Information flow diagram of the sweetening process after using assumptions (1: absorber, 2-1: heater, 2-2: precooler, 3: stripper, 4: solvent pump, 5: solution makeup, and 6: after-cooler).

**Figure 7:**
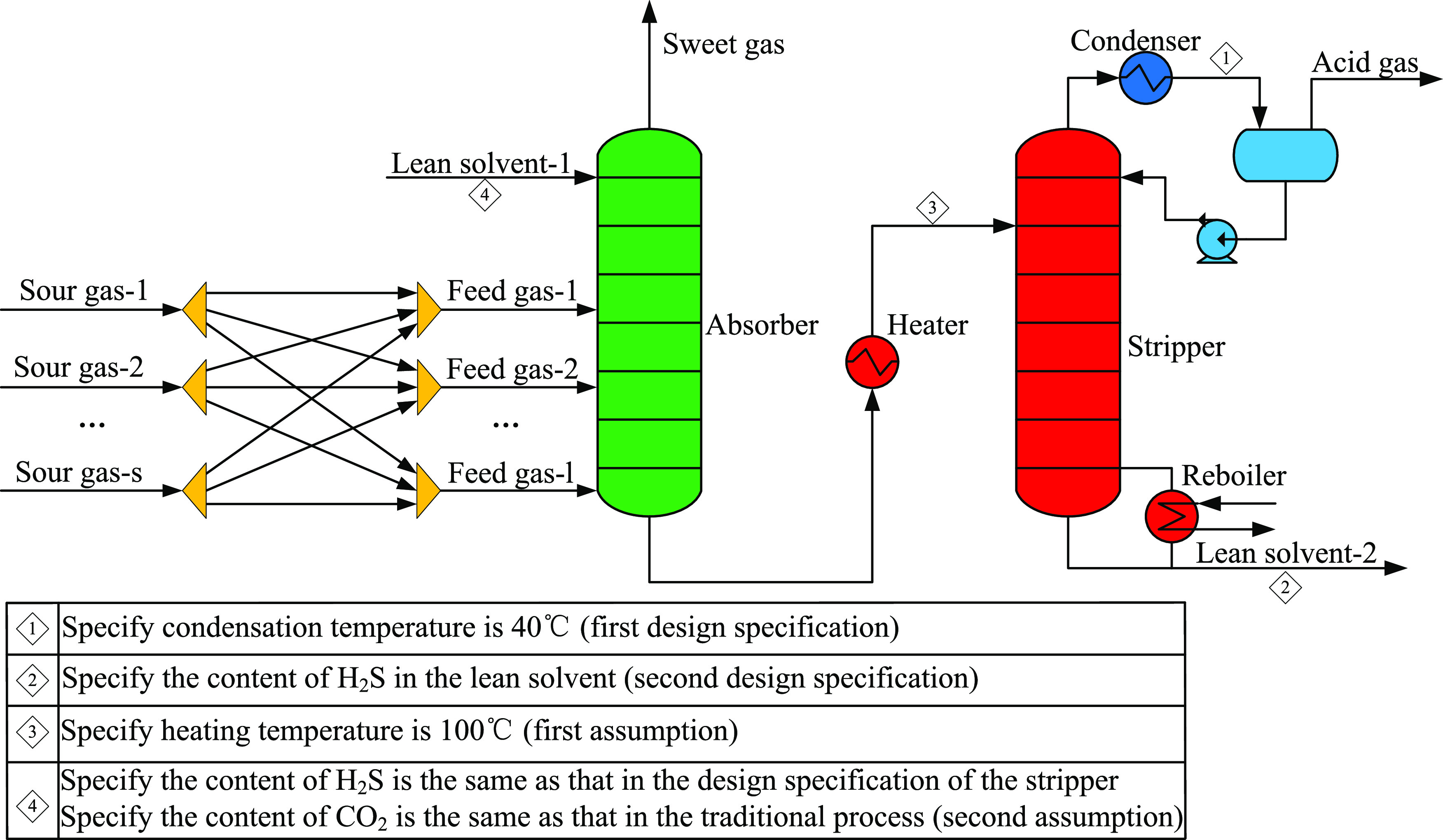
Simplified simulation process.

Through replacement of the cross heat exchanger with a heater and a cooler, the entire system contains only one loop; the information flow diagram is shown in Figure 6. According to the second assumption, the composition of the lean solvent will be completely specified, and the loop structure of the entire system can be broken, which greatly guarantees the convergence of the process and reduces the optimization time. In addition, the operating cost of the cooler and pump is negligible and not included in the objective function, so the final simplified simulation process is shown in Figure 7. With the simplified process, the single simulation time can be reduced by 30–70%; meanwhile, the entire optimization time can also be significantly reduced.

### 4.4 Hybrid Simulation Platform

Optimization of the multiple gas feed sweetening process is a strongly nonlinear problem due to rigorous rate-based equations and thermodynamic properties with ionic characteristics as well as considerable amount of decision variables and built-in design specifications. In Aspen HYSYS, the built-in algorithm of the column relaxes the accuracy of the solution to ensure the convergence of the model as much as possible, usually resulting in small numerical noise. This numerical noise can be large enough to prevent the calculation of accurate derivatives and make the derivative-based optimization algorithms unreliable.^37^ These problems can be avoided through metaheuristic algorithms for black-box optimization, where optimal solutions can be obtained through population-based search techniques without requiring any derivative information.^55^

In this work, a particle swarm optimization (PSO) algorithm was adopted because of its robust convergence in the optimization of complicated and nonlinear problems,^56^ the relative ease of implementation, and requiring only a few tuning parameters.^57^ The PSO is a new swarm intelligence algorithm proposed by Kennedy and Eberhart.^58^ In this model, each particle’s own state is composed of a set of position vectors and a velocity vector, which, respectively, represent the feasible solution of the problem and its direction of motion in the search space. The particle constantly learns the group optimal solutions and individual optimal solution, updates its own velocity and position, and realizes the global optimal search. Therefore, the particle position and velocity update equation is the core of the PSO algorithm (see eqs 37 and 38)
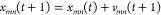
37
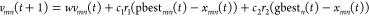
38where *w* is the inertial weight, which indicates the influence of the historical velocity information of the particle on the current velocity. When *w* is large, the particle has a strong global exploration ability; when *w* is small, the particle has a strong local convergence performance. Therefore, a linear adjustment strategy is adopted for *w* (eq 39)
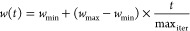
39

To combine the simulator with the PSO algorithm, a hybrid simulation platform was used. Aspen HYSYS v11 is automated by MATLAB R2018a as the external solver, which programmatically runs Aspen HYSYS as a frontend. On the one hand, Aspen HYSYS can strictly calculate the absorption and desorption process, and the built-in algorithm has good convergence for a single column. On the other hand, MATLAB programmatically controlled black-box functions inside Aspen HYSYS and takes all relevant decisions to attain the optimal design with the PSO algorithm.

**Figure 8:**
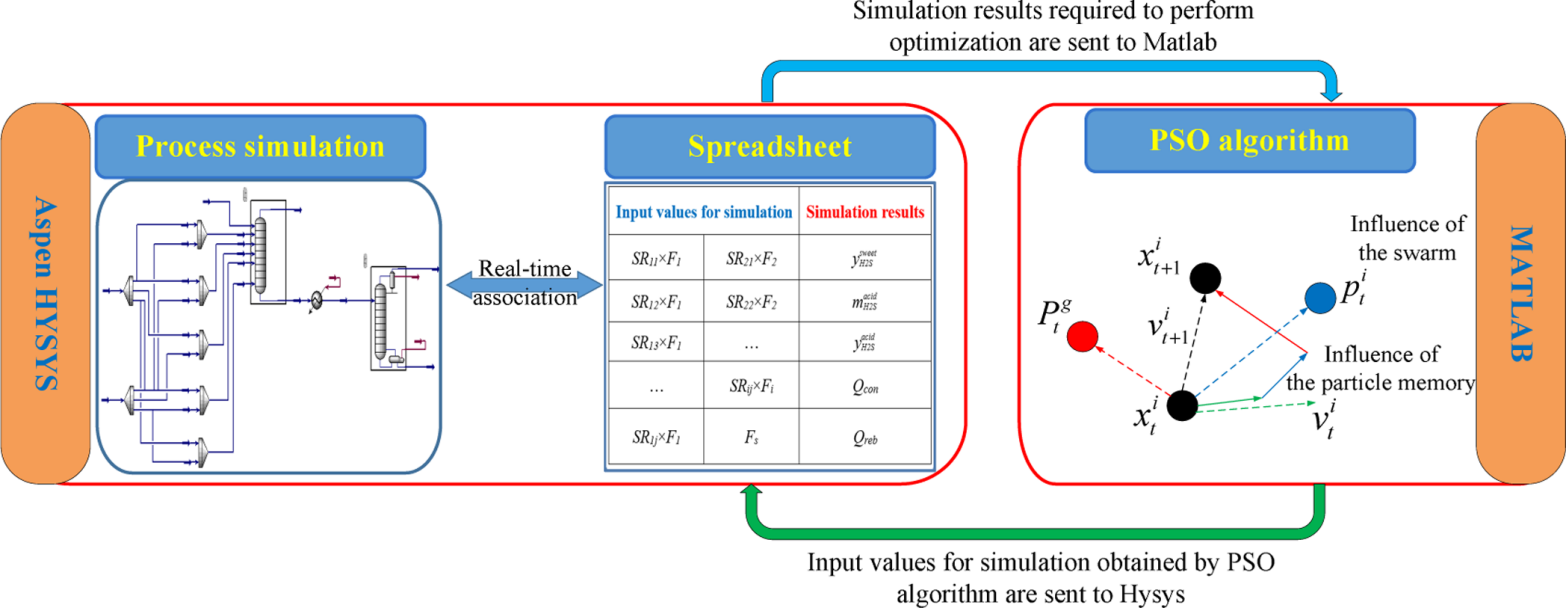
HYSYS–MATLAB interface.

A connection between MATLAB and Aspen HYSYS can be established through a Component Objective Model (COM) in ActiveX, which allows direct two-way communication between Aspen HYSYS and MATLAB. As shown in Figure 8, the input and output data in the unit operation can be related with the cell data of the built-in spreadsheet in real time in Aspen HYSYS, and MATLAB can directly access the spreadsheet. In the optimization, the decision variables are obtained from the PSO, namely, the split ratio of each raw gas (*SR*_*ij*_) and the flow rate of solution (*F*_s_), where the split ratio of each raw gas is multiplied by its flow rate (*F*_*i*_) to obtain the flow rate of each raw gas to each feed stage, and then these flow rates and the flow rate of solution are transferred from MATLAB to HYSYS for simulation. After simulation, the flow rate of H_2_S in acid gas (*m*_H_2_S_^acid^), purity of H_2_S in acid gas (*y*_H_2_S_^acid^), duties of the condenser and reboiler (*Q*_con_ and *Q*_reb_), and content of H_2_S in sweet gas (*y*_H_2_S_^sweet^) by HYSYS are sent back to MATLAB for the calculation of the PSO fitness function, which performs the optimization. In addition, MATLAB can also control the active and reset status of unit operation and effectively diagnose fault. Through the connection between MATLAB and Aspen HYSYS, it can not only perform high-fidelity model calculations but also obtain optimal solutions by the metaheuristic algorithm to meet the needs of conceptual design. All simulation runs and executed algorithms are performed on a PC with an i5-1035G1 CPU and 8 GB RAM.

### 4.5 Optimization Framework

**Figure 9:**
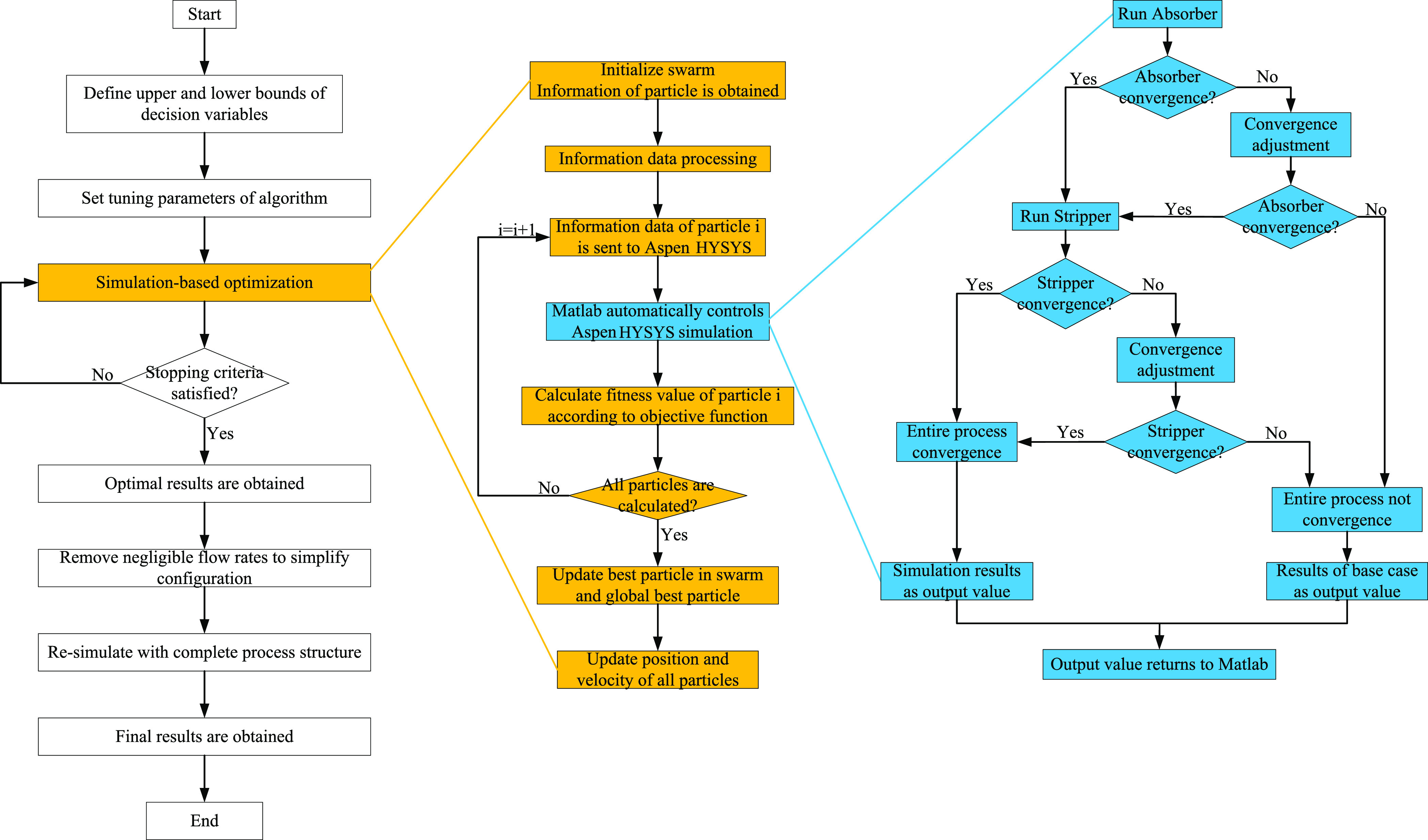
Optimization framework.

A simulation-based optimization framework is demonstrated in Figure 9. When the upper and lower bounds of decision variables and tuning parameters of the algorithm are determined, the simulation-based optimization starts, and the optimal results are obtained after the stopping criteria are satisfied. In a subsequent step, streams with negligible flow rates can be removed to simplify the optimal configuration. The resulting simplified configuration should then be resimulated with a complete process structure (i.e., with a cross heat exchanger and a circulating solvent stream) to confirm the final results.

**Figure 10:**
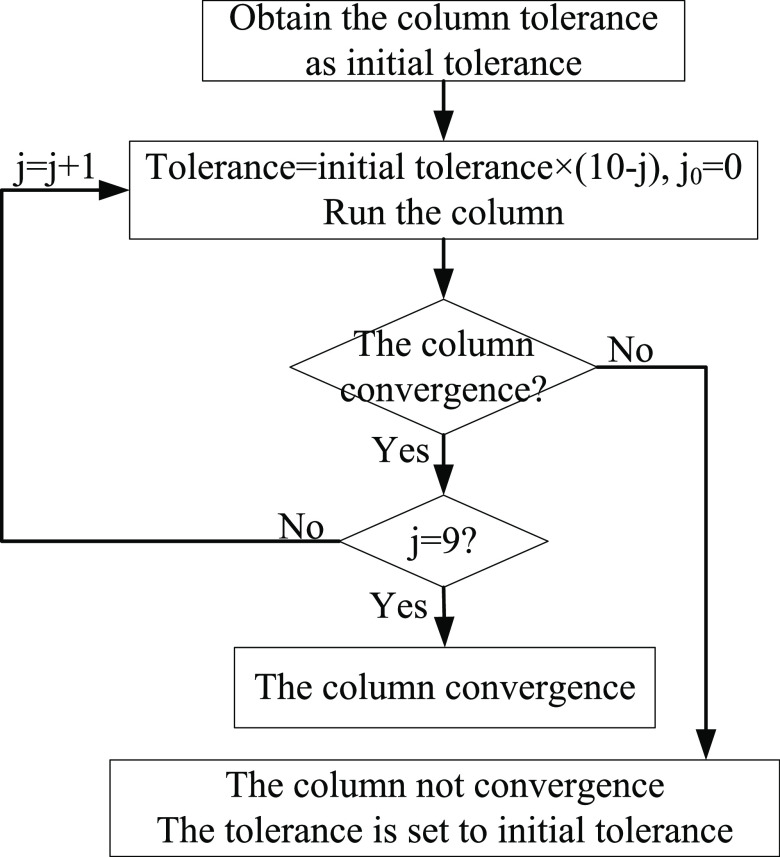
Convergence adjustment process.

For the simulation-based optimization, the PSO algorithm first initializes all particles and obtains the information of each particle, which is the split ratio of each sour gas and the amount of the solvent. Since the information data is continuous variable generated under unconstrained conditions, the value of split ratio is normalized and then the information data is sent to Aspen HYSYS. To ensure the continuity and improve the convergence of the simulation, a sequential module simulation method is used to control the simulation sequence of each column through MATLAB. This method can control the simulation sequence by switching the operation status of the absorber and stripper. Only when the absorber converges, the simulation of the stripper starts. When a column does not converge, a convergence adjustment is performed. The most effective adjustment method is to give the column a reasonable initial value to promote its convergence. In this work, a stepwise approach is used to promote convergence. The convergence adjustment process is shown in Figure 10. First, the tolerance of the column is relaxed to promote convergence and then the tolerance is gradually reduced, and the simulation result after convergence is used as the initial value of the next simulation to stepwise adjust its convergence. Finally, when all columns converge, the simulation results are used as the output value. If there is still no convergence after adjustment, we terminate the simulation and use the simulation results of the conventional process (i.e., base case) as the output value to ensure the continuity of optimization. The above method can effectively avoid the automatic termination of the calculation due to the nonconvergence of a column, and the sequential module simulation method can be used to locate the nonconvergent column and perform convergence adjustment, which improves the convergence of the entire process.

## 5 Case Study

A refinery in Shandong Province, China, where two sulfur-containing dry gases are produced by two catalytic cracking plants, is taken for demonstration. The two dry gases are sent to the same plant for sweetening treatment with 30 wt % MDEA solution, and it is finally ensured that the H_2_S content in the sweet gas is less than 20 ppm_v_. The information of each dry gas and absorber and stripper is shown in Tables 3 and 4, respectively.

**Table 3:** Information of Two Dry Gases

	dry gas-1	dry gas-2
pressure (MPaG)	1.18	1.15
temperature (°C)	40	40
flow rate (kmol/h)	515.4	158.3
molar composition (%)		
H_2_S	2.18	9.47
CO_2_	9.17	4.68
H_2_	31.32	28.24
C_1_	23.16	20.01
C_2_	8.91	5.92
C_2_=	11.70	7.71
C_3_	0.08	0.07
C_3_=	0.47	0.43
C_4_	0.05	0
air	12.96	23.47

**Table 4:** Operating Conditions and Equipment Information of Absorber and Stripper

	absorber	stripper
number of trays	10	15
inside diameter (m)	1.2	0.8
tray type	valve	valve
tray spacing (m)	0.5	0.5
solvent feed stage from top, tray	1	3
solvent feed temperature (°C)	45	100
top pressure (kPa)	1101	150

### 5.1 Optimization Results

In the conventional process, the two dry gases are mixed first and then sent to the bottom of the absorber. In the optimization process, feed gas is allowed to enter from the 6th tray to the bottom of the absorber. When the feed stage of the feed gas is above the 6th tray, the number of trays used for absorption will be too small, which will lead to a significant increase in the amount of solvents and energy consumption. Hence, each dry gas contains five split ratios (SR_*m*,*n*_) ranging from 0 to 1 and corresponding to five feed stages. The subscript *m* can be 1 or 2, respectively, representing dry gas-1 or dry gas-2, while subscript *n* can be 1–5, respectively, representing the 6th to 10th tray. In addition, the amount of solvent (*F*_s_) also needs to be optimized to ensure the content of H_2_S in sweet gas is less than 20 ppm_v_. The upper bound of the amount of solvent is determined by the worst case where all of the feed gases are sent to the 6th tray of absorber. Since the proposed process can reduce the amount of the solvent, the lower bound is set to be 0.9 times that of the conventional process.

For PSO, the larger the number of particles, the larger the searching space of the algorithm, which means it is more likely to find a better solution, but this in turn would require more running time. A guidance rule is that the number of particles generally needs to be a bit more than the number of decision variables in the optimization. In the case study, the number of decision variables in the optimization is 11, and 15 particles are selected according to the tradeoff between the performance and the complexity of the PSO algorithm. The maximum number of iterations of the PSO algorithm is set to 200. By multiple test experiments, it is found that after 200 times, the optimization results tend to be stable for our cases. When the number of iterations is exceeded or the deviation of the results of 100 consecutive iterations does not exceed 10^–5^, the iteration will be terminated. For this example, the entire optimization time is about 4.5 h. The method of convergence adjustment was used in the optimization, and there is no nonconvergence during the whole process. Without the proposed method, there will be no less than 10 nonconvergences in the entire optimization. Due to the use of the stochastic algorithm, the strategy of multiple optimizations should be introduced to guarantee the optimality of the solution. In this work, five optimizations were considered for each case study, and the best optimization result was selected. All optimization results and models are provided in the Supporting Information (see Sections S2 and S4).

**Figure 11:**
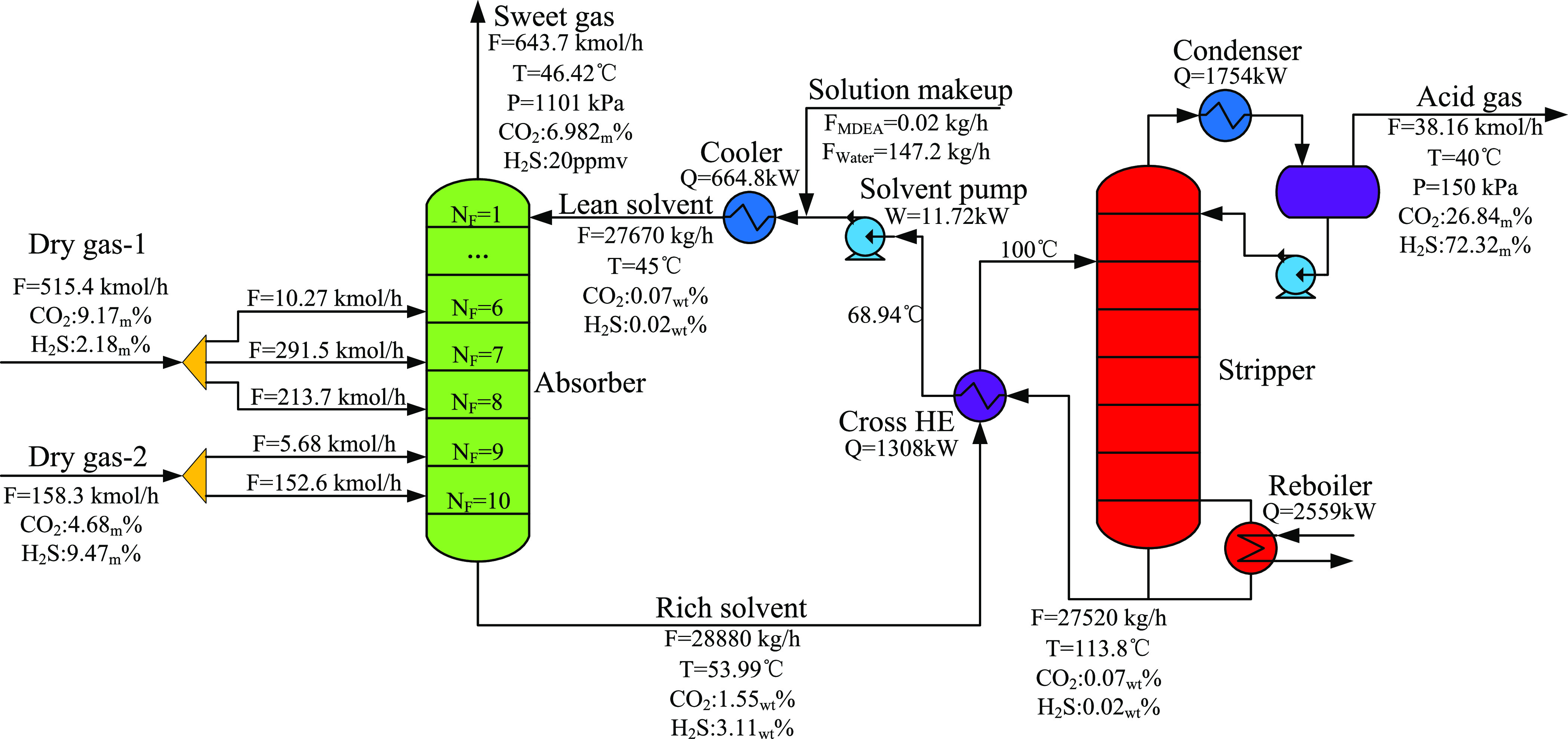
Flowsheet of the optimized multiple gas feed sweetening process (Δ*P*_H_2_S_ = 0.72 $/h).

The optimized results of decision variables are shown in Table 5. After removing the streams with a negligible flow rate, the final optimization process is shown in Figure 11. There are five feed streams for the absorber. Dry gas-1 is divided into three streams and, respectively, sent to the 6th, 7th, and 8th trays, 98.02% of which enters the 7th and 8th trays. Dry gas-2 is divided into two streams and, respectively, sent to the 9th and 10th trays, 96.4% of which enters the 10th tray (i.e., the bottom of the absorber). Since the reaction of CO_2_ and the MDEA solution is slow, the absorption of CO_2_ is similar to physical absorption, while the absorption of H_2_S is obviously promoted by chemical reactions, and the temperature and concentration distribution in the absorber also have a significant impact on it. Dry gas-1 with higher CO_2_/H_2_S ratio is sent to the 6th–8th trays; this can not only reduce the temperature in the absorber improving the absorption of H_2_S but also effectively reduce the absorption amount of CO_2_ and ultimately increase the purity of H_2_S in acid gas. Due to the competitive relationship in absorption between CO_2_ and H_2_S, the reduction of the CO_2_ absorption amount can decrease the concentration of CO_2_ in the liquid phase in the absorber, thereby further improving the absorption of H_2_S and reducing the amount of the lean solvent and energy consumption.

**Table 5:** Optimized Results of Decision Variables

decision variable	value	decision variable	value
SR_11_	0.0199	SR_21_	2.15 × 10^–4^
SR_12_	0.5648	SR_22_	3.20 × 10^–3^
SR_13_	0.414	SR_23_	1.67 × 10^–3^
SR_14_	8.83 × 10^–4^	SR_24_	0.0357
SR_15_	4.32 × 10^–4^	SR_25_	0.9592
*F*_s_ (kg/h)	27 670		

The optimization results of conventional and proposed processes are shown in Table 6. Detailed stream data of conventional and proposed processes is listed in the Supporting Information (see Section S3). Compared with the conventional process, the proposed process has achieved the effect of improving the purity of H_2_S and reducing energy consumption. The purity of H_2_S is increased from 69.28 to 72.32%, and the product value is increased accordingly. In addition, the duty of the reboiler is reduced from 2750.38 to 2558.89 kW. Eventually, the operating cost is decreased by 7.07%, and the overall economic benefit is increased by 73.36%.

**Table 6:** Optimization Results

	conventional process	proposed process
purity of H_2_S in acid gas (%)	69.28	72.32
duty of reboiler (kW)	2750.38	2558.89
duty of condenser (kW)	1869.94	1754.47
duty of cooler (kW)	781.87	664.75
product value ($/h)	127.08	129.03
operating cost ($/h)	113.49	105.47
economic benefit ($/h)	13.59	23.56

### 5.2 Process Flexibility Analysis

The economic benefits of the sweetening process are mainly determined by product prices and utility costs. For the conventional process, there is a lack of adjustment means to respond to market fluctuations. The advantage of the new process is that it can flexibly adjust the product purity and plant energy consumption to respond to market fluctuations. Therefore, to study the effect of product price on the process, the cost of utilities is fixed, and Δ*P*_H_2_S_ is increased from 0.72 $/t to 4.5 $/t.

**Table 7:** Optimized Results of Decision Variables

Δ*P*_H_2_S_ ($/t)	0.72	1.5	2.5	3.5	4.5
SR_11_	0.0199	0.2566	0.3879	0.5494	0.6152
SR_12_	0.5648	0.4224	0.3751	0.3506	0.3838
SR_13_	0.4140	0.2275	0.2369	0.0820	8.87e-5
SR_14_	8.83 × 10^–4^	0.0935	0	0.0181	1.34 × 10^–4^
SR_15_	4.32 × 10^–4^	0	1.00 × 10^–4^	6.00 × 10^–5^	7.80 × 10^–4^
SR_21_	2.15 × 10^–4^	5.89 × 10^–4^	2.41 × 10^–3^	6.60 × 10^–4^	0
SR_22_	3.20 × 10^–3^	0	2.65 × 10^–4^	0	8.89 × 10^–3^
SR_23_	1.67 × 10^–3^	0	0.0528	0	0.0898
SR_24_	0.0357	8.44 × 10^–3^	0.0182	0.0848	0.3274
SR_25_	0.9592	0.9910	0.9263	0.9146	0.5739
F_s_ (kg/h)	27670	27770	27780	27790	27990

**Figure 12:**
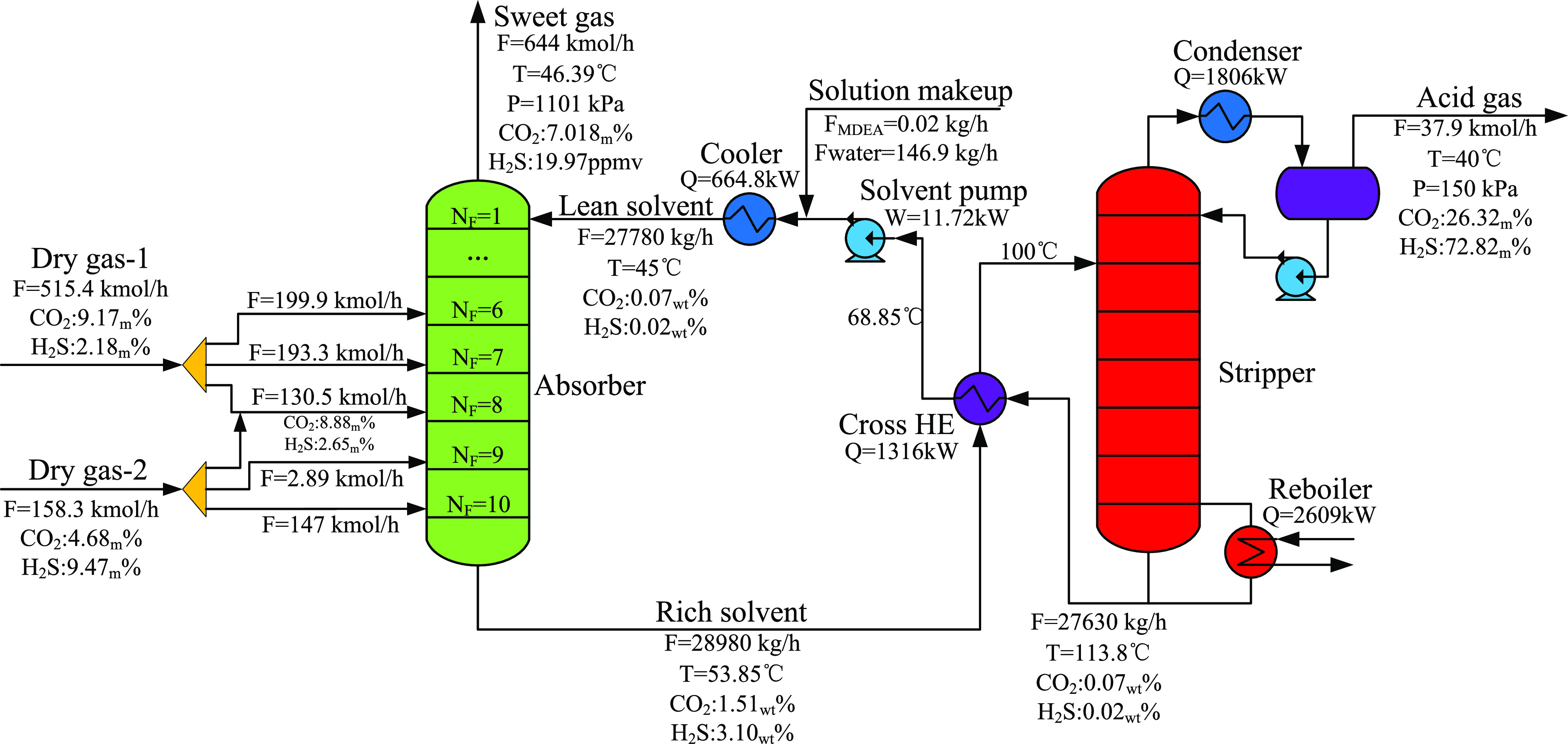
Flowsheet of the optimized multiple gas feed sweetening process (Δ*P*_H_2_S_= 2.5 $/t).

**Figure 13:**
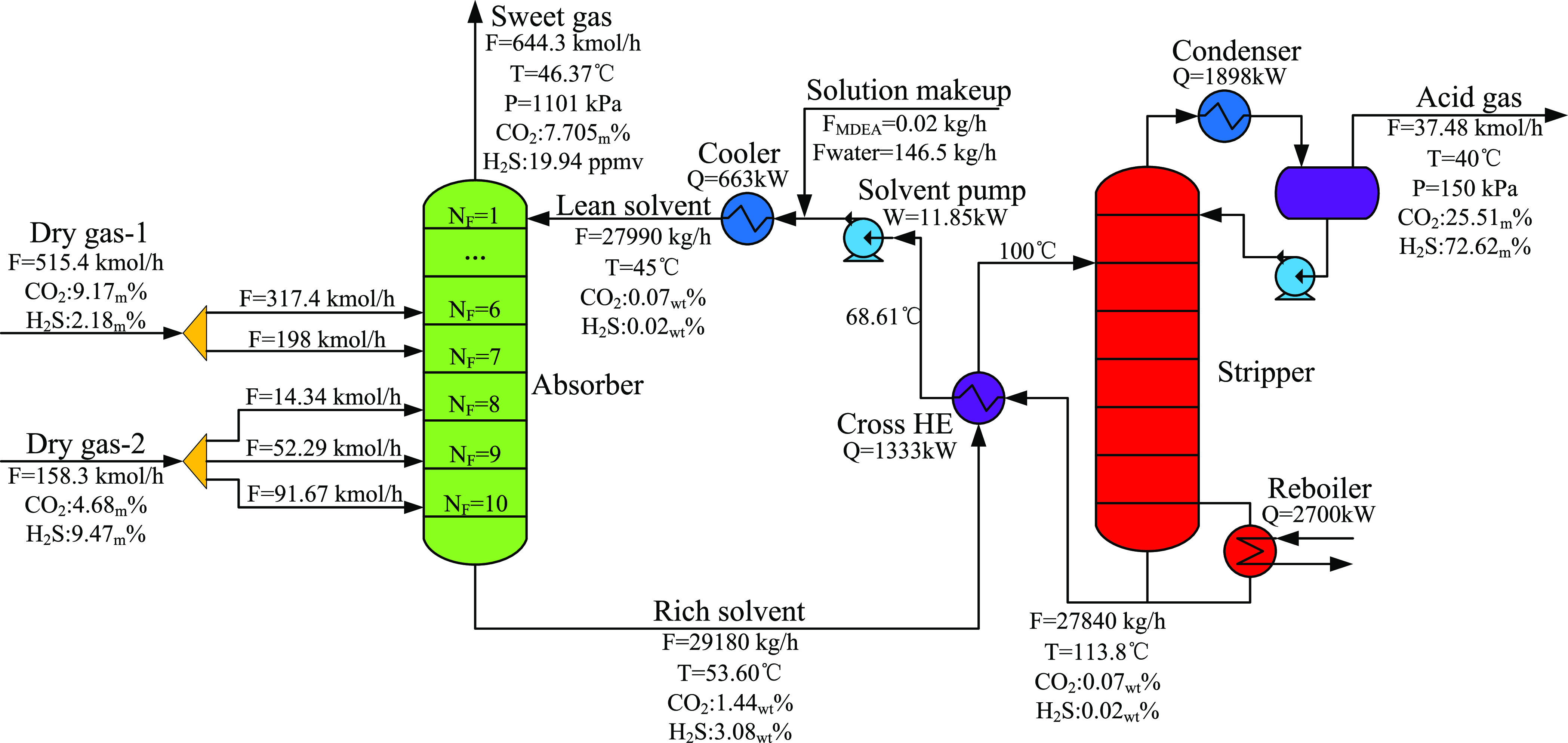
Flowsheet of the optimized multiple gas feed sweetening process (Δ*P*_H_2_S_ = 4.5 $/t).

The optimization results of each decision variable and process are shown in Tables 7 and 8, respectively. It can be seen that the proposed process can flexibly adjust the product purity and energy consumption by optimizing the split ratio of each dry gas. When Δ*P*_H_2_S_ increases from 0.72 $/t to 4.5 $/t, the product purity increases from 72.32% to 73.62%, and the duty of the reboiler increases from 2559 kW to 2700 kW, which is still lower than that of the conventional process, and the economic benefits of the entire plant increase. Optimization flowsheets of the new process for Δ*P*_H_2_S_ being 2.5 $/t and 4.5 $/t, respectively, are displayed in Figures 12 and 13. When the added value of the product increases, the proposed process will increase the feed flow rate of the upper tray to obtain higher purity of H_2_S at the expense of increased energy consumption.

**Table 8:** Optimization Results

Δ*P*_H_2_S_ ($/t)	0.72	1.5	2.5	3.5	4.5
purity of H_2_S in acid gas (%)	72.32	72.36	72.82	73.17	73.62
duty of reboiler (kW)	2558.89	2575.57	2608.83	2638.79	2700.33
duty of condenser (kW)	1754.47	1769.82	1805.64	1837.57	1898.47
duty of cooler (kW)	664.75	671.97	664.77	662.59	663.03
product value ($/h)	129.03	130.71	133.83	137.46	142.07
operating cost ($/h)	105.47	106.18	107.54	108.78	111.32
economic benefit ($/h)	23.56	24.53	26.29	28.68	30.75

**Table 9:** Compositions (mol %) of Sour Gases from Different Sources

	natural gas					biogas		syngas
component	Ekofisk, Norway	Panhandle, USA	Wustrow, Germany	Süd-Oldenburg, Germany	Tenguiz, USSR	anaerobic digestion	landfill gas	coal gasification
CH_4_	85	73.2	43	77	42	60–70	35–65	0–5
C_2_+	12.5	11.5	0.7	0.1	39			
CO_2_	2	0.3	0.3	8	2.6	30–40	15–40	5–15
H_2_S	0.001			8	16	0–0.4	0–0.01	0–0.01
N_2_	0.4	14.3	56	7	0.8	0–0.5	15	
H_2_								25–30
CO								30–60
NH_3_						0.01	0.0005	
He		0.7	0.04					

### 5.3 Sensitivity Analysis of Feedstocks

The quality of sour gases is determined by their composition, and it plays an important role in the influence of energy consumption and product purity. The compositions of sour gases from different sources^59^ are shown in Table 9. The contents of H_2_S and CO_2_ in sour gases from different sources vary greatly. Therefore, to explore the impact of the composition of the sour gas on the proposed process, a comparison group is set up, the information is shown in Table 10. The sour gas in the comparison group was taken from the works of Muhammad^60^ and Abdulrahman.^61^ The difference in the ratio of CO_2_/H_2_S between the two sour gases is smaller. Since the separation of the stripper in the comparison group is more difficult, the amount of the gas phase in the column is larger, so the inner diameter of the stripper is adjusted to 1 m.

**Table 10:** Information of Sour Gases in the Comparison Group

items	sour gas-1	sour gas-2
flow rate (kmol/h)	515.4	158.3
molar composition %		
H_2_S	1.72	5.38
CO_2_	4.13	4.48
H_2_		
CH_4_	86.92	63.35
C_2_	3.93	13.9
C_3_	0.93	6.03
C_4_	0.55	3.8
C_4_+	0.44	2.95
H_2_O	1.22	
N_2_	0.16	0.11
O_2_		

The optimization results of each decision variable and process are shown in Tables 11 and 12, respectively. Due to the small difference in the CO_2_/H_2_S ratio of the two sour gases, the adjustment space for the concentration distribution in the absorber is reduced, so the reduction in energy consumption and the increase in the purity of H_2_S are correspondingly reduced, and the economic benefit is only increased by 24.06% compared with the conventional process. Through the above analysis, the greater the difference in the composition of sour gases, the more obvious the advantages obtained by the proposed process.

**Table 11:** Optimized Results of Decision Variables in Comparison Groups

decision variable	value	decision variable	value
SR_11_	4.06 × 10^–5^	SR_21_	0
SR_12_	0.1996	SR_22_	1.14 × 10^–2^
SR_13_	0.3508	SR_23_	0
SR_14_	0.1971	SR_24_	0
SR_15_	0.2525	SR_25_	0.9886
*F*_s_ (kg/h)	22 180		

**Table 12:** Optimization Results of Comparison Groups

	conventional process	proposed process
purity of H_2_S in acid gas (%)	74.65	75.32
duty of reboiler (kW)	2428.99	2361.93
duty of condenser (kW)	1829.26	1778.28
duty of cooler (kW)	578.09	450.35
product value ($/h)	86.55	86.84
operating cost ($/h)	100.39	97.34
economic benefit ($/h)	–13.84	–10.51

## 6 Conclusions

In this study, a simulation-based optimization method was presented to maximize the economic benefits of the multiple gas feed sweetening process. A series of design specifications are imposed on the Aspen HYSYS model based on surrounding environmental conditions, process design guarantee, and common standard practice. Moreover, a series of reasonable assumptions were introduced to simplify the process, eliminate the loop structure, and greatly reduce the optimization time. In addition, due to the complexity of the mass transfer model and the thermodynamic model considering the ion characteristics, a strategy of controlling the simulation sequence along with a stepwise adjustment method to adjust the nonconvergent column by MATLAB was adopted to ensure the convergence of the simulation during the optimization process.

A case study was carried out to demonstrate the proposed approach. With the above method, the times of nonconvergence can be reduced to zero. The results show that the optimal process can increase the purity of H_2_S from 69.28% to 72.32%, while the energy consumption is reduced by 7.07%, and the overall economic benefit is increased by 73.36%. Through the analysis of the proposed process, it is found that the proposed process can flexibly adjust the process parameters to respond to market fluctuations. Furthermore, for the larger difference in the composition of sour gases, the proposed process is more economical.

## 7 Future Research

In the future work, we mainly focus on the following two aspects:

(1) The current optimization method will generate too complicated configurations for industrial implementation. The complete preallocations will lead to a complex pipeline network and control system in actual applications. Therefore, to introduce the complexity index to characterize the complexity of the sour gas preallocation and using multiobjective optimization to tradeoff the process complexity and economic benefits is one of the priority works.
(2) The other important work is to simultaneously optimize the sweetening process and the downstream H_2_S processing unit, such as the sulfur recovery unit, NaHSO_4_ unit, and thiourea unit. This will avoid misleading caused by the inappropriate price of H_2_S.

## Supporting Information Available

(ZIP)
Comparison of simulation modes, results of each optimization, and stream data of the conventional process and proposed process (PDF)

## Notations

*a*: penalty factor
*a*^I^: interfacial area for mass transfer [m^2^]
*c*_1_: acceleration factor
*c*_2_: learning factor
*C*_op_: operating cost [$/h]
*F*: feed molar flow rate [kmol/s]
*F*_s_: circulating rate of the solvent
*G*: Gibbs free energy [J/mol]
gbest: optimal position of swarm
*H*: enthalpy [J/kmol]
*H̅*: partial enthalpy [J/kmol]
*h*: heat transfer coefficient [J/m^2^·K·s]
*k*_R1_: reaction rate constant of R1
*k*_R2_: reaction rate constant of R2
*K*_r_: equilibrium reaction constant
*K*: phase equilibrium constant
*k*: mass transfer coefficient [m/s]
*L*: liquid molar flow rate [kmol/s]
*m*_H_2_S_^acid^: flow rate of H_2_S in acid gas [kg/h]
*N*: mass transfer rate [kmol/s]
*nc*: number of components
*P*: pressure [Pa]
*P*_*H*_2_*S*_: price of H_2_S [$/t]
Δ*P*_H_2_S_: increased value of price when the purity of H_2_S increase per 1% compared to the basic purity [$/t]
*P*_H_2_S_^base^: price of the basic purity of H_2_S [$/t]
*P*_u_: price of utilities [$/kJ]
Profit: economic benefits [$/h]
pbest: optimal position of individual particle
*Q*: heat input to a stage [J/s]
*Q*_u_: heat duties of utilities [kJ/h]
*Q*_con_: heat duty of condenser [kJ/h]
*Q*_reb_: heat duty of reboiler [kJ/h]
*q*: heat transfer rate [J/s]
***R***: inverse of the mass transfer coefficient matrix [s/kmol]
R: gas constant (8.3144 J/mol K)
*r*: reaction rate [kmol/m^3^·s]
*r*_1_: random numbers between 0 and 1
*r*_2_: random numbers between 0 and 1
SR: split ratio
*T*: temperature [K]
*t*: generations
*V*: vapor molar flow rate [kmol/s]
*V*_pro_: product value [$/h]
*v_mn_*: velocity of *n*th dimension of the *m*th particle
*w*: inertial weight
*x*: liquid mole fraction
*x*_mn_: position of the *n*th dimension of the *m*th particle
*y*: vapor mole fraction
*y*_H_2_S_^acid^: purity of H_2_S in acid gas
*y*_H_2_S_^base^: basic purity of H_2_S
*y*_H_2_S_^sweet^: content of H_2_S in sweet gas
*z*: electric charge number
**Greek Letters**
Δ*ϕ*^E^: driving force caused by electric potential
***Γ***: matrix of thermodynamic factors
*Γ*: liquid flow per unit length of perimeter [kg/m·s]
*δ_i_*_,_*_k_*: Kronecker delta: 1 if *i* = *k*, 0 otherwise
ρ̅: molar density [kmol/m^3^]
φ: fugacity coefficient
ψ: electric potential
γ: activity coefficient
ν: stoichiometric coefficient
∑: fixing the mole fractions of all other components except the *n*th while evaluating the differentiation
**Superscripts**
*F*: feed
*f*: film
*I*: interface
*L*: liquid
sweet: sweet gas
target: target value
*V*: vapor
**Subscripts**
e: educt
for: forward reaction
*i*,*k*,*m*: component
*j*: stage
*n*: last component
*P*: product
*rev*: reverse reaction
*r*: reaction index
*t*: total
