# Supplementary material for: Simulation-Based Optimization of a Multiple Gas Feed Sweetening Process
Source: ACS Omega. 2022 Jan 13;7(3):2690–705. doi: 10.1021/acsomega.1c05193 (PMC8793078; doi:10.1021/acsomega.1c05193)
Supplement: Supplementary file 2 — ao1c05193_si_002.pdf [file ao1c05193_si_002.pdf]

Supporting information
(For publication)

Simulation-Based Optimization of a Multiple Gas Feeds Sweetening Process

Weixuan Zhu, Haotian Ye, Yang Yang, Xiong Zou, Hongguang Dong**

School of Chemical Engineering, Dalian University of Technology, Dalian 116024, China

*Email: htye@dlut.edu.cn (Haotian Ye); hgdong@dlut.edu.cn (Hongguang Dong)

S1. Comparison of simulation modes

There are two simulation modes in Aspen Hysys to simulate the absorber and stripper, namely advanced mode and efficiency mode. Both modes apply the two-film theory to calculate the mass and heat transfer process. The difference is that the efficiency mode simplifies the rate-based model (for example, efficiency mode does not account for resistance to heat transfer and assumes the liquid phase is in chemical equilibrium), and uses the simplified rate-based model to first calculate the Murphree efficiency of H_2S and CO_2 (assuming the Murphree efficiency of other components is 1), and then uses the equilibrium-based model and Murphree efficiency to calculate the absorption or distillation process. Therefore, the efficiency model can effectively improve the convergence of the simulation, but it will also reduce the accuracy of the simulation.

In the sweetening process, the use of appropriate simulation modes for the absorber and stripper is the key to optimization, and the selection needs to weigh the convergence and accuracy of the simulation mode. Table S1 shows the comparison results of absorber and stripper use different simulation modes under different composition of feed gas (the feed gas only contains H_2S, CO_2 and methane, and the content of methane is 10 $_{m}$%), where the flow rate of feed gas is 500 kmol/h, the content of MDEA in the lean solvent is 30 $_{wt}$%, the operating pressures of the absorber and stripper are 1000 kPa and 150 kPa, respectively, and the diameters are 1.2 m and 1.5 m, the feed of the stripper is the rich solvent obtained by using the advanced mode to simulate the absorber with different feed gas.

Table S1 The comparison results of absorber and stripper use different simulation modes

<table>
<tr><td rowspan="2">Feed gas</td><td colspan="3">Content of H_2S/$_m$%</td><td>10</td><td>8</td><td>6</td><td>4</td><td>2</td></tr>
<tr><td colspan="3">Content of CO_2/$_m$%</td><td>0</td><td>2</td><td>4</td><td>6</td><td>8</td></tr>
<tr><td rowspan="7">Absorber</td><td colspan="3">Flow rate of solvent/(kg/h)</td><td>32860</td><td>29310</td><td>25580</td><td>21680</td><td>17550</td></tr>
<tr><td rowspan="6">Composition of sweet gas</td><td rowspan="3">H_2S ppm$_v$</td><td>Advanced mode</td><td>19.63</td><td>19.09</td><td>19.86</td><td>20.10</td><td>20.16</td></tr>
<tr><td>Efficiency mode</td><td>12.40</td><td>44.16</td><td>75.79</td><td>70.23</td><td>52.15</td></tr>
<tr><td>Relative deviation/%</td><td>-36.8</td><td>131.4</td><td>281.7</td><td>249.5</td><td>158.7</td></tr>
<tr><td rowspan="3">CO_2 $_m$%</td><td>Advanced mode</td><td>0.00</td><td>1.67</td><td>3.31</td><td>4.93</td><td>6.55</td></tr>
<tr><td>Efficiency mode</td><td>0.00</td><td>1.63</td><td>3.25</td><td>4.86</td><td>6.45</td></tr>
<tr><td>Relative deviation/%</td><td>/</td><td>-2.10</td><td>-1.75</td><td>-1.58</td><td>-1.50</td></tr>
<tr><td rowspan="6">Stripper</td><td colspan="2" rowspan="3">Duty of reboiler kW</td><td>Advanced mode</td><td>6316</td><td>4471</td><td>3320</td><td>2529</td><td>1927</td></tr>
<tr><td>Efficiency mode</td><td>6376</td><td>4234</td><td>3127</td><td>2343</td><td>1672</td></tr>
<tr><td>Relative deviation/%</td><td>0.95</td><td>-5.30</td><td>-5.81</td><td>-7.35</td><td>-13.23</td></tr>
<tr><td colspan="2" rowspan="3">Duty of condenser kW</td><td>Advanced mode</td><td>5382</td><td>3647</td><td>2611</td><td>1942</td><td>1469</td></tr>
<tr><td>Efficiency mode</td><td>5442</td><td>3410</td><td>2418</td><td>1756</td><td>1214</td></tr>
<tr><td>Relative deviation/%</td><td>1.11</td><td>-6.50</td><td>-7.39</td><td>-9.58</td><td>-17.36</td></tr>
</table>

It can be found from Table S1 that under the same flow rate of solvent, the composition of the sweet gas obtained by the two simulation modes of the absorber is quite different, and the content of CO_2

in the sweet gas calculated by the efficiency mode is lower than that of the advanced mode. When there is CO_2 in the feed gas, the calculated content of H_2S in the sweet gas is higher than that of the advanced mode. If the content H_2S in the sweet gas is ensured to be the same, the content of CO_2 calculated by the efficiency mode will be even lower. Because the absorber is the core of the entire process, accurate calculation of composition is the key to process optimization, and there are no design specifications for the absorber, both simulation modes can effectively converge using their own built-in algorithm. Therefore, the absorber uses advanced mode. When the content of impurities in the lean solvent at the bottom of the stripper is a fixed value, the duty of reboiler or condenser calculated by the two simulation modes differs greatly at the content of CO_2 in feed gas is high. Because the content of H_2S in the feed gas in the study is relatively high, and the change trend of the duty of reboiler and condenser is the same under the calculation of the two simulation modes, which will not affect the optimized process structure. However, due to the design specifications in the simulation of the stripper to ensure the temperature at the top of the column and the composition of the lean solvent at the bottom of the column, the use of advanced mode will cause the simulation of the stripper to be difficult to converge, so the efficiency mode is used in consideration of the convergence.

The industrial data[1] and calculation results are shown in Table S2, where the absorber uses advanced mode and stripper uses efficiency mode. It can be seen that except the flow rate of H_2S in sweet gas, the deviations of other data are all within 5%. Since the flow rate of H_2S is trace, a large deviation is inevitable, the selection of simulation modes for absorber and stripper are appropriate.

Table S2 Comparison of calculation results and industrial data

Parameters	Industrial data	Calculation results	Relative deviation /%
	Sweet gas		
Flow rate of CO_2/(kmol/h)	676	656.123	-2.94
Flow rate of H_2S/(kmol/h)	0.6	0.41	-31.89
Temperature/℃	-	73.28	-
Pressure/bar	66.5	66.5	0
Flow rate/(kmol/h)	29835	29792.9	-0.14
	Acid gas		
Flow rate of CO_2/(kmol/h)	456.6	472.79	3.55
Flow rate of H_2S/(kmol/h)	169.6	168.97	-0.37
Temperature/℃	57	57	0
Pressure/bar	2	2	0
Flow rate/(kmol/h)	686.66	706.29	2.86

S2. Optimization results

Due to the use of stochastic algorithms, the results of each optimization are different. Therefore, each case study is optimized five times during optimization, and the best result is selected. The optimization results of each case study are shown in Table S3-S8. Table S3-S7 is the optimization results when ΔP_{H2S} is 0.72, 1.5, 2.5, 3.5 and 4.5 \$/t, Table S8 is the optimization results of the comparison group. Since the range of each split ratio during optimization is 0 to 1, but SR_{11} to SR_{15} and SR_{21} to SR_{25} need to meet the constraint of adding to 1, so when Matlab sends data to Aspen Hysys, the spilt ratio will be normalized.

Table S3 Optimization results at ΔP_{H2S} is 0.72 \$/t

Number	1	2	3	4	5
SR_{11}	0.7583	0.9615	0.7280	0.7490	0.0267
SR_{12}	0.4838	0.7730	0.8724	0.3065	0.7583
SR_{13}	0.5015	0.8734	0.5236	0.7311	0.5559
SR_{14}	0.0044	0.8801	0.1381	0.0972	0.0012
SR_{15}	0.1770	0.0000	0.1752	0.1716	0.0006
SR_{21}	0.0110	0.0000	0.0015	0.0005	0.0002
SR_{22}	0.0161	0.0000	0.0123	0.0011	0.0026
SR_{23}	0.0324	0.0000	0.5398	0.0180	0.0014
SR_{24}	0.6089	0.3611	0.7920	0.0581	0.0289
SR_{25}	1.0000	0.8941	0.9919	0.6412	0.7771
F_s/(kg/s)	7.7667	7.7373	7.8550	7.6935	7.6861
Objective function	22.4943	23.7113	21.8148	24.3122	25.2041

Table S4 Optimization results at ΔP_{H2S} is 1.5 \$/t

Number	1	2	3	4	5
SR_{11}	0.1480	0.4199	0.5793	0.9060	0.5894
SR_{12}	1.0000	0.7103	0.6022	0.7604	0.9702
SR_{13}	0.7817	0.2670	0.2350	0.6277	0.5226
SR_{14}	0.2003	0.0481	0.2537	0.2583	0.2148
SR_{15}	0.0350	0.0267	0.0009	0.6509	0.0000
SR_{21}	0.0000	0.0000	0.0000	0.0000	0.0004
SR_{22}	0.0128	0.0000	0.0057	0.0000	0.0000
SR_{23}	0.0011	0.0000	0.0023	0.0008	0.0000
SR_{24}	0.3733	0.7800	0.5902	0.0041	0.0057
SR_{25}	0.8411	0.8000	0.8984	0.9889	0.6684
F_s/(kg/s)	7.7073	7.6608	7.6768	7.7935	7.7139
Objective function	25.9328	25.5530	25.1763	25.9466	26.1919

Table S5 Optimization results at ΔP_{H2S} is 2.5 $/t

Number	1	2	3	4	5
SR_{11}	0.5510	0.6483	0.7926	0.7612	0.8293
SR_{12}	0.8877	0.9755	0.5553	0.6392	0.8019
SR_{13}	1.0000	0.1476	0.3898	0.5752	0.5065
SR_{14}	0.5524	0.0001	0.3865	0.0681	0.0000
SR_{15}	0.0262	0.0671	0.0000	0.0055	0.0002
SR_{21}	0.0025	0.0000	0.0002	0.0004	0.0021
SR_{22}	0.0000	0.0000	0.0000	0.0000	0.0002
SR_{23}	0.0011	0.0162	0.0000	0.0411	0.0471
SR_{24}	0.0020	0.4289	0.0176	0.8573	0.0162
SR_{25}	0.9786	1.0000	0.8961	0.8529	0.8249
F_s/(kg/s)	7.6806	7.5967	7.6222	7.6767	7.7167
Objective function	27.6269	27.7646	27.5983	26.2464	27.9341

Table S6 Optimization results at ΔP_{H2S} is 3.5 $/t

Number	1	2	3	4	5
SR_{11}	0.6126	0.6224	0.8220	0.7964	0.6077
SR_{12}	0.6681	0.6917	0.3658	0.6617	0.3878
SR_{13}	0.5331	0.7627	0.2668	0.0060	0.0907
SR_{14}	0.0008	0.0321	0.0222	0.0152	0.0200
SR_{15}	0.0204	0.0000	0.0050	0.0366	0.0000
SR_{21}	0.0036	0.0000	0.0000	0.0006	0.0007
SR_{22}	0.0000	0.0173	0.0121	0.0010	0.0000
SR_{23}	0.0006	0.0249	0.0311	0.0452	0.0000
SR_{24}	0.3120	0.5071	0.1824	0.4346	0.0856
SR_{25}	0.9615	0.9813	0.7838	0.9047	0.9237
F_s/(kg/s)	7.5970	7.6507	7.6325	7.6060	7.7194
Objective function	30.0557	29.1506	30.2640	30.0600	30.3187

Table S7 Optimization results at ΔP_{H2S} is 4.5 $/t

Number	1	2	3	4	5
SR_{11}	0.9435	0.5839	0.7929	0.8542	0.6803
SR_{12}	0.7546	0.7400	0.3741	0.6711	0.4244
SR_{13}	0.4363	0.0005	0.0001	0.8434	0.0001
SR_{14}	0.0080	0.0014	0.0020	0.0113	0.0001
SR_{15}	0.0061	0.0011	0.0006	0.0000	0.0009
SR_{21}	0.0049	0.0000	0.0025	0.0000	0.0000
SR_{22}	0.0052	0.0016	0.0366	0.0024	0.0155
SR_{23}	0.0136	0.0067	0.2449	0.0237	0.1564
SR_{24}	0.0153	0.6563	0.6329	0.8530	0.5703
SR_{25}	0.9890	0.9139	0.9071	0.9888	1.0000
F_s/(kg/s)	7.6027	7.5855	7.6559	7.6526	7.7750
Objective function	31.2430	32.1653	31.3358	29.0634	32.3898

Table S8 Optimization results of comparison group

Number	1	2	3	4	5
SR_{11}	0.1226	0.0461	0.0351	0.2974	0.0001
SR_{12}	0.4931	0.8300	0.5754	0.7362	0.4765
SR_{13}	0.1106	0.7862	1.0000	0.7888	0.8375
SR_{14}	1.0000	0.4423	0.4211	0.3333	0.4706
SR_{15}	0.1255	0.9348	0.0419	0.7693	0.6028
SR_{21}	0.0015	0.0000	0.0000	0.0000	0.0000
SR_{22}	0.0292	0.0943	0.0000	0.0000	0.0114
SR_{23}	0.0000	0.0289	0.0167	0.0000	0.0000
SR_{24}	0.0975	0.2438	0.4349	0.0010	0.0000
SR_{25}	0.8713	0.9481	0.8067	0.9653	0.9886
F_s/(kg/s)	6.1936	6.3144	6.2490	6.1755	6.1611
Objective function	-9.7650	-12.2828	-11.3470	-9.9881	-9.3962

S3. Stream data

Flow charts of the conventional process and the proposed process are shown in Figures S1 and S2, and the corresponding stream data are shown in Tables S9 and S10. In order to reduce the influence of the number of components on the optimization process, and considering that the influence of inert components on the absorption process is negligible, all C2 and heavier components and air are treated as ethane according to the molecular weight.

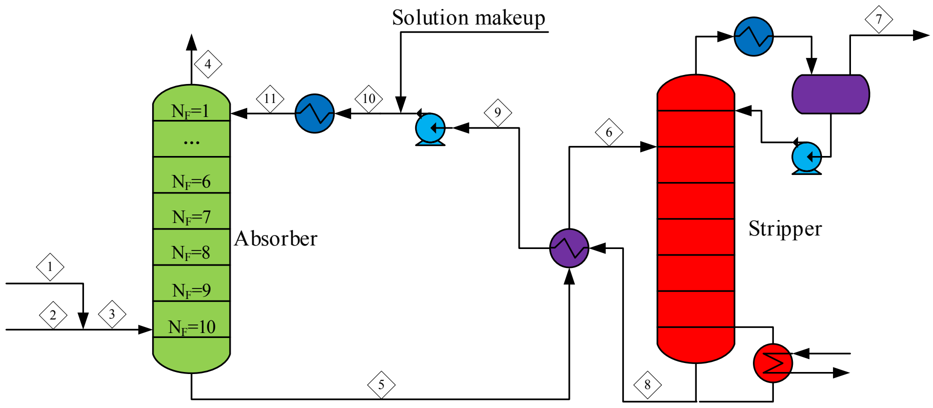

Figure S1 Flowchart of the conventional process

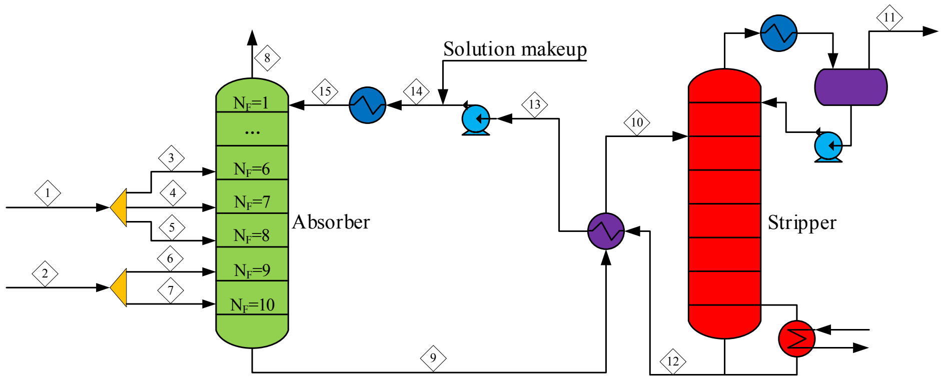

Figure S2 Flowchart of the proposed process

Table S9 Stream data of the conventional process

Stream number	1	2	3	4	5	6
Temperature/℃	40.00	40.00	39.99	46.24	53.82	100.00
Pressure/kPa	1301	1301	1301	1101	1101	1101
Flowrate/(kmol/h)	515.40	158.30	673.70	642.05	1319.47	1319.47
Mole fraction/%						
CO_2	9.17	4.68	8.11	6.76	0.90	0.90
H_2S	2.18	9.47	3.89	0.00	2.00	2.00
Methane	23.16	20.01	22.42	23.51	0.01	0.01
Hydrogen	31.32	28.24	30.60	32.08	0.01	0.01

H_2O	0.00	0.00	0.00	0.96	91.13	91.13
MDEA	0.00	0.00	0.00	0.00	5.94	5.94
Ethane	34.17	37.60	34.98	36.68	0.01	0.01

Table S9 Stream data of the conventional process (continued)

Stream number	7	8	9	10	11
Temperature/℃	40.00	113.79	68.80	68.98	45.00
Pressure/kPa	150	155	155	1301	1301
Flowrate/(kmol/h)	39.83	1279.64	1279.64	1287.82	1287.82
Mole fraction/%					
CO_2	28.33	0.04	0.04	0.04	0.04
H_2S	65.81	0.01	0.01	0.01	0.01
Methane	0.17	0.00	0.00	0.00	0.00
Hydrogen	0.33	0.00	0.00	0.00	0.00
H_2O	5.00	93.81	93.81	93.85	93.85
MDEA	0.00	6.13	6.13	6.09	6.09
Ethane	0.35	0.00	0.00	0.00	0.00

Table S10 Stream data of the proposed process

Stream number	1	2	3	4	5	6	7	8
Temperature/℃	40.00	40.00	40.00	40.00	40.00	40.00	40.00	46.42
Pressure/kPa	1301	1301	1301	1301	1301	1301	1301	1101
Flowrate/(kmol/h)	515.40	158.30	10.27	291.46	213.67	5.68	152.62	643.72
Mole fraction/%								
CO_2	9.17	4.68	9.17	9.17	9.17	4.68	4.68	6.98
H_2S	2.18	9.47	2.18	2.18	2.18	9.47	9.47	0.00
Methane	23.16	20.01	23.16	23.16	23.16	20.01	20.01	23.46
Hydrogen	31.32	28.24	31.32	31.32	31.32	28.24	28.24	32.00
H_2O	0.00	0.00	0.00	0.00	0.00	0.00	0.00	0.97
MDEA	0.00	0.00	0.00	0.00	0.00	0.00	0.00	0.00
Ethane	34.17	37.60	34.17	34.17	34.17	37.60	37.60	36.58

Table S10 Stream data of the proposed process (continued)

Stream number	9	10	11	12	13	14	15
Temperature/℃	53.99	100.00	40.00	113.81	68.95	69.13	45.00
Pressure/kPa	1101	1101	150	155	155	1301	1301
Flowrate/(kmol/h)	1173.97	1173.97	38.16	1135.81	1135.81	1144.00	1144.00
Mole fraction/%							
CO_2	0.87	0.87	25.50	0.04	0.04	0.04	0.04
H_2S	2.25	2.25	68.69	0.01	0.01	0.01	0.01
Methane	0.00	0.00	0.15	0.00	0.00	0.00	0.00
Hydrogen	0.01	0.01	0.30	0.00	0.00	0.00	0.00
H_2O	90.93	90.93	5.00	93.81	93.81	93.86	93.86

MDEA	5.93	5.93	0.00	6.13	6.13	6.09	6.09
Ethane	0.01	0.01	0.36	0.00	0.00	0.00	0.00

S4. Illustration of model files

The model files (base case .hsc, modified raw gas .hsc, conventional process .hsc, proposed process .hsc) are all based on Aspen Hysys v11, which need to be opened in the Aspen Hysys v11 or higher. The model files (main_base_case .m, main_raw_gas .m, objective_base_case .m, objective_raw_gas .m,) are all based on Matlab. The description of each model file is as follows:

Conventional process .hsc: the model file is used to simulate the conventional process.

Proposed process .hsc: the model file is used to simulate the proposed process when the ΔP_{H2S} is 0.72 $/t.

base case .hsc: the model is automated by Matlab to optimize the process, where the feed gas are dry gas-1 and dry gas-2.

modified raw gas .hsc: the model is automated by Matlab to optimize the process of the comparison group.

main_base_case .m, objective_base_case .m: the model file is used to optimize the process of dry gas-1 and dry gas-2 as the feed gas.

main_raw_gas .m, objective_raw_gas .m: the model file is used to optimize the process of the comparison group.

References

(1) Abd, A. A.; Naji, S. Z.; Barifcani, A. Comprehensive evaluation and sensitivity analysis of regeneration energy for acid gas removal plant using single and activated-methyl diethanolamine solvents. *Chin. J. Chem. Eng.* **2020**, *28*, 1684-1693.
